# Ethnobotanical assessment of antidiabetic medicinal plants in District Karak, Pakistan

**DOI:** 10.1186/s12906-024-04462-w

**Published:** 2024-04-24

**Authors:** Amina Nazar, Muhammad Adnan, Syed Majid Shah, Ahmed Bari, Riaz Ullah, Akash Tariq, Nisar Ahmad

**Affiliations:** 1 Department of Botany, Kohat University of Science and Technology, Kohat, 26000 Pakistan; 2 Department of Pharmacy, Kohat University of Science and Technology, Kohat, 26000 Pakistan; 3 Department of Pharmaceutical Chemistry, College of Pharmacy King Saud University, Riyadh, Saudi Arabia; 4 Department of Pharmacognosy, College of Pharmacy King Saud University, Riyadh, Saudi Arabia; 5 Xinjiang Key Laboratory of Desert Plant Roots Ecology and Vegetation Restoration, Xinjiang Institute of Ecology and Geography, Chinese Academy of Sciences, Urumqi, 830011 China

**Keywords:** Diabetes mellitus, Ethnobotany, Formulation, Medicinal plants, Quantitative study

## Abstract

**Background:**

Diabetes is a leading health disorder and is responsible for high mortality rates across the globe. Multiple treatment protocols are being applied to overcome this morbidity and mortality including plant-based traditional medicines. This study was designed to investigate the ethnomedicinal status of plant species used to treat diabetes in District Karak, Pakistan.

**Materials and methods:**

A semi-structured survey was created to collect data about traditionally used medicinal plants for diabetes and other ailments. The convenience sampling method was applied for the selection of informants. The collected data was evaluated through quantitative tools like frequency of citation (FC), relative frequency of citation (RFC), informant consensus factor** (**FIC), fidelity level (FL), and use value (UV).

**Results:**

A total of 346 local informants were selected for this research. Out of them, 135 participants were men and 211 participants were women. Overall 38 plant species belonging to 29 plant families were used to treat diabetes. The most dominant plant family was Oleaceae having 11 species. Powder form (19%) was the most recommended mode of preparation for plant-based ethnomedicines. Leaves (68%) were the most frequently used parts followed by fruit (47%). The highest RFC was recorded for *Apteranthes tuberculata* (0.147). The maximum FL was reported for *Apteranthes tuberculata* (94.4) and *Zygophyllum indicum* (94.11) for diabetes, skin, and wounds. Similarly, the highest UV of (1) each was found for *Brassica rapa*, *Melia azedarach,* and *Calotropis procera*. Based on documented data, the reported ailments were grouped into 7 categories. The ICF values range between 0.89 (diabetes) to 0.33 (Cardiovascular disorders).

**Conclusion:**

The study includes a variety of antidiabetic medicinal plants, which are used by the locals in various herbal preparations. The species *Apteranthes tuberculata* has been reported to be the most frequently used medicinal plant against diabetes. Therefore, it is recommended that such plants be further investigated *in-vitro* and *in-vivo* to determine their anti-diabetic effects.

## Background

Plants are the essential components of the planet Earth and are considered an excellent source of medicine. They are utilized in the treatment or prevention of a wide variety of diseases like diabetes, cancer, and cardiovascular problems [1]. Globally, diabetes *mellitus* is considered one of the leading causes of death as well as a top health condition [2]. A common metabolic disorder, diabetes *mellitus* occurs when there is insufficient supply of insulin (Type I) or when the body is resistant to insulin (Type II) [3]. Type II diabetes is also characterized by two significant conditions resulting from defective insulin secretion or reduced insulin sensitivity (insulin resistance) [4]. A report presented by the World Health Organization indicates that the diabetic population is possible to increase up to 300 million or more in 2025 [5]. Approximately 536.6 million people worldwide suffer from diabetes (diagnosed or undiagnosed) in 2021 according to the International Diabetes Federation and by 2045, this number will grow by 46% to 783.2 million [6]. There are several methods of treating diabetes including insulin, allopathic homeopathic, and traditional herbal medicines [7, 8].

According to International Diabetes Federation an estimated 26.7% of Pakistani adults affected by diabetes in 2022, and the number may reach 33 million approximately [9]. If awareness programs and proper treatment are not adopted, Pakistan may have double the number of diabetic patients by 2040 [10] and it is expected to reach 37.1 million by 2045 [11]. Additionally, diabetes was found to be more prevalent in urban areas (15.1%) than in rural areas (1.6%) [12]. A study reported that in rural areas, women's tolerance for glucose is 10.9% and men's is 6.9%, but in urban areas, it is 14.2% and women's is 6.3% [13].

The rural population of Pakistan relies significantly on their conventional herbal system for diabetic problems because the cost of allopathic prescription treatment for diabetes is too high [14]. According to research by the WHO, 855 traditional medicines use crude plant extracts, and more than 86% of people in underdeveloped countries depend on traditional remedies like herbs for their everyday requirements [15]. Plants with antidiabetic properties are commonly used in folk medicines. In ethnobotanical studies, around 800 plants are thought to have anti-diabetic effects [16]. In addition to these 800 plants, *Momordica charantia*, *Pterocarpus marsupium*, and *Trigonella foenum-graecum* have been reported to benefit type 2 diabetes patients [17]. Laboratory experiments have shown that around 343 plants have been tested for blood glucose [18]. The control of glucose metabolism is greatly influenced by natural plant products with a variety of distinct chemical components, such as phenolic compounds, flavonoids, terpenoids, alkaloids, glycosides, and coumarins [19]. Multiple herbs have exposed antidiabetic activity when reviewed using streptozotocin-induced diabetic rats [20]. Natural substances known as antioxidants help diabetes patients by scavenging different types of free radicals and decreasing the harm carried on by oxidative stress [21].

District Karak has diverse environmental conditions with unique flora. The people who live in this region are well-versed in the uses of plants. It not only reflects the unique identity of a community but also provides accessible and affordable healthcare solutions, particularly in areas where modern medical resources are scarce. As traditional healers often maintain close ties with their communities, they provide a holistic approach to health, focusing on not only physical but also mental and spiritual well-being. Their expertise, grounded in local knowledge of plants, herbs, and environmental factors, contributes to the diagnosis and treatment of ailments specific to the region. Local inhabitants use different plants for the treatment of different diseases like diabetes, cardiovascular, gastrointestinal, skin, and rheumatoid traditionally [22].

Studies on ethnobotany focus on the complex interactions between native people and plants, including traditions and cultural beliefs related to different applications. This research supports the collection of essential ethnobotanical data from indigenous people, such as herbalists, to conserve traditional knowledge of disease diagnosis and plant species utilized in folk medicine. In addition, their methods of planning and management, as well as the indigenous people's sociocultural heritage for future generations. The present research work is designed to summarize data about ethnobotanical data of plant species used against diabetes. This study aimed to have a long-term date of plants used traditionally by local inhabitants of southern regions of KPK, Pakistan. The area was selected based on rich sources of medicinal wild local plants, traditional uses of medicines by local people, and easily accessible areas.

## Materials and methods

### Study area

**Fig. 1:**
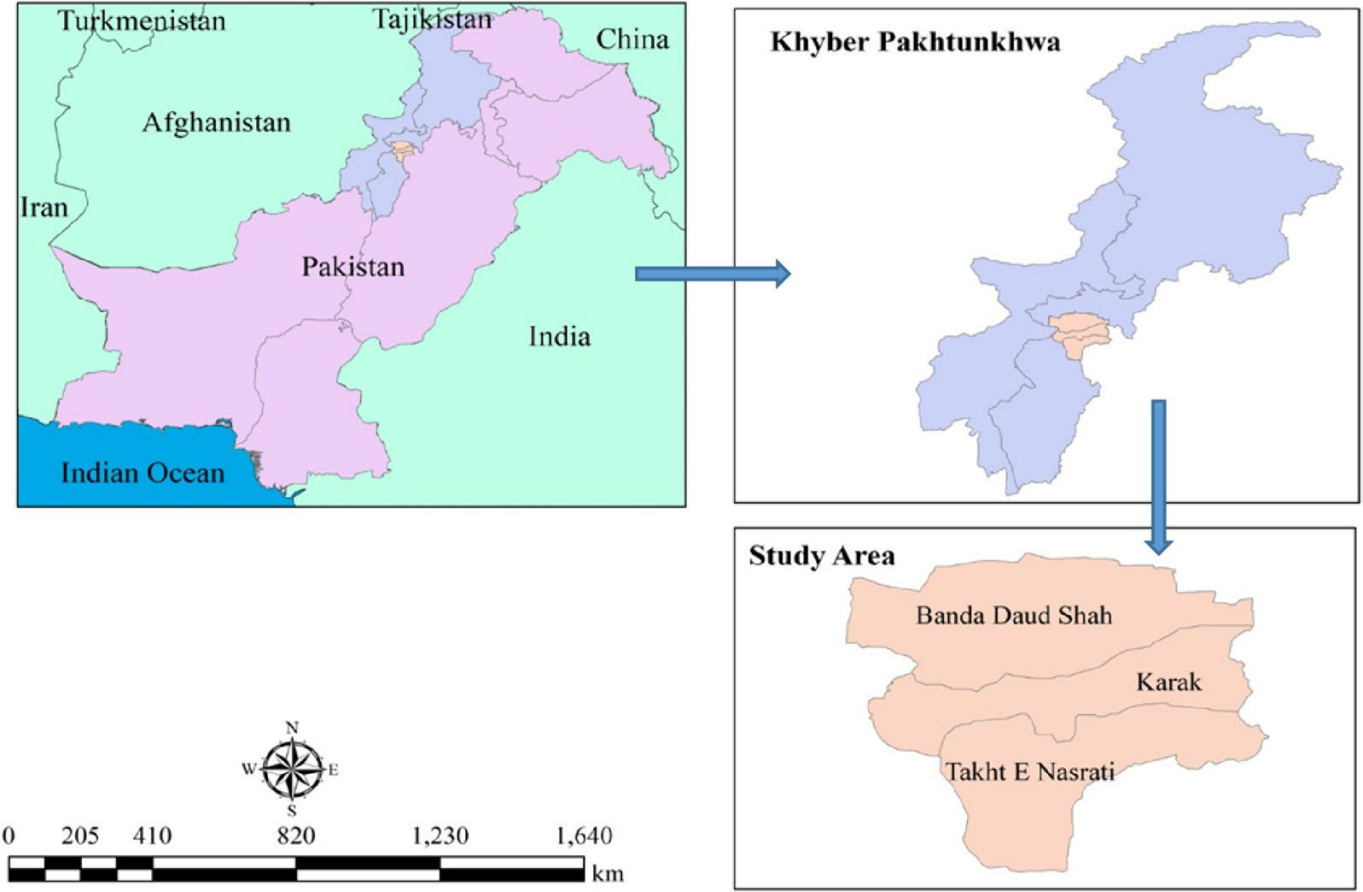
Geographical location of the study area. Indicating three Tehsils (Takht-e-Nasrati, Karak, and Banda Daud Shah) where the study was conducted in District Karak, Khyber Pakhtunkhwa province, Pakistan

The current survey was carried out in District Karak, Pakistan (Fig. 1). The district Karak is situated in the south of KPK between latitudes 70-40° and 71-30°N and longitudes 32-48° and 33-23°E It includes both rural and urban areas with a total size of 600 Km^2^[23]. Small mountains make up the geography of the Karak district, and these mountains generally move from east to west. The climate during summer is hot with temperatures ranging from 40-45 ^0^C. Sand storms are common in Tehsil Takht-e-Nasrati. Most areas in the district are arid. The area is rich in flora. Mostly it comprises xerophytes. Common species of the area are *Acacia nilotica, Acacia arabica*, *Calotropis procera*, *Peganum harmala,* and *Citrullus colocynthis*. Cultivated includes Wheat, Sorghum, Peanut, and Gram. The district is home to a variety of fauna like quail, crane, black and brown. It's a popular place to hunt quails and fowl (Batair). In Karak, many natural resources have been found. The salt mines were an important source of salt for the Indo-Pak subcontinent throughout the British period and were well-known in antiquity. Uranium, gas, and oil were found more recently. Makori, Noshpa Banda, and Gurguri are the three towns where oil and gas deposits have been discovered. Being rich in wild herbs, people have sufficient knowledge about medicinal plants and their use against different ailments.

### Data collection and field survey

Data on ethnomedicines was collected from September 2021 to February 2022. The data was collected in the Pashtu (native language) and then translated into English through a semi-structured questionnaire. The first section of the questionnaire includes the demographics of the informants, age, gender, and educational status. While remaining part of the questionnaire consists of knowledge of medicinal plants to treat diabetes and other ailments. Mainly concentrating on the native name of the medicinal plant, other additional ingredients, remedy preparation, which part of the plant is used, and the mode of administration and dosage information for children and adults. A convenience sampling method was used to collect information regarding medicinal plants used against diabetes traditionally. Participants in the study were acknowledged as well informed about local medicinal plants and had long-standing interactions with the local flora and ecosystem. Our survey's primary respondents, rather than conventional healers or professionals, were common people with local plant knowledge who learned it orally from their elders [24].

Code of Ethics of the International Society of Ethnobiology (ISE) was strictly followed while conducting the survey [25]. Before each interview, each participant verbally agreed. A description of the study's purpose and the subject matter was given to each participant. The convenience sampling method was applied to select the interviewers using the Cochran formula (1977) for sample size. *n*^0^=z2⋅p⋅ (1−p) e2. After calculation from the Cochran formula, a total of 790 primary participants were visited to know about their knowledge about the traditional usage of medicinal plants. Out of these, 346 respondents were considered for their knowledge related to the plants against diabetes.

In the first instance, local people medically diagnosed with diabetes were identified and designated as participants for this study. They were questioned about the plants, which are traditionally used against diabetes along with other ailments. This study primarily focused on medicinal plants used to cure diabetes.

### Collection, identification, and preservation of medicinal plants

Plant species collected from the study area were identified by Dr.Ghazala Nawaz, Assistant Professor, Department of Botany, Kohat University of Science and Technology, Kohat, voucher numbers and deposition numbers were given e.g. BOT-KHS-90/2023-001 (BOT-KHS-90 represents voucher number(s) while 2023-001 is the deposition number). The specimens were then submitted to the Herbarium at the Department of Botany, Kohat University of Science and Technology, Pakistan. For specimen validation, the www.floraonline.org/taxon/wfo-0001020013 was accessed.

### Data quality assurance

Each primary participant was contacted at least three times to confirm the information they had provided for data confirmation. Only validated and related information was subject to further analytical processes. Moreover, the authors were skilled in collecting medicinal plants from the area as well as gathering remedy formulation-related information, their uses, pointing out missing information, and replication material to maintain data quality.

### Ethnobotanical indices

Frequency of citation (FC), relative frequency of citation (RFC), Species Use Value (UV), Fidelity level (FL), and Informant consensus factor (ICF), were among the quantitative indices used to analyze the ethnobotanical data.

#### Frequency of citation (FC)

FC is the number of primary participants who described using each monoherbal recipe for ethnomedicinal purposes [26].

#### Relative frequency of citation (RFC)

Relative frequency of citation (RFC) indicates the importance of each species locally within a study region [27]. N is the total number of informants in the survey, and FC is the number of primary participants who cited a helpful species. Using the previously discussed formula, RFC is determined. When the RFC value is 0, it indicates that fewer primary participants have found that monoherbal formulation to be helpful, and when the RFC value is 1, it indicates that more survey primary participants have found that monoherbal formulation to be helpful (Table 2) [28].

$$RFC=FC /N (0 < RFC < 1)$$

### Use Value (UV)

Plant utilization standards are possessed and followed [29].

$$\text{Used value}=\text{quantity of utilization}/{\text{N}}$$

The N represents the total number of primary participants while U represents the amount of utilization average collected from each source for known species of plant and the Use value conveys the quantifiable sum of qualified purpose of species of plant.

#### Fidelity level (FL%)

Fidelity level is the ratio between the number of primary participants who independently mention one use of a plant species and the total number of primary participants who initially mention all uses of that plant species proposed by [30]. It is calculated as follows: Np is the number of primary participants who reported the usage of a species; N is the total number of primary participants who mentioned all the uses of that species; and N is multiplied by 100. When a plant species has a high FL for particular uses, the local population prefers that species for that use. The species with high FL authenticates its uniqueness to treat a disease [31].

$$FL\% =\frac{Np }{N}\times 100$$

Np =number of primary participants citing the use of the plant species for the treatment of a disease

N = total number of primary participants citing the species for disease.

#### Informant consensus factor (ICF)

To estimate the usage variability of medicinal plants by local informants, ICF was used [32]. It is a consensus between local primary participants for the treatment of a disease or disease category. ICF was calculated by following the formula;

$$ICF=\frac{Nur-Nt}{Nur-1}$$

N_ur_ = total number of use citations for each disease category

N_t_ = total number of species listed in that category

The value of ICF ranges from 0 to 1. A higher value means there are well-defined criteria for medicine in the areas for a specific disease. A low value indicates that plants are not preferred and there is no exchange of information about their use [33].

### Literature review

**Fig. 2:**
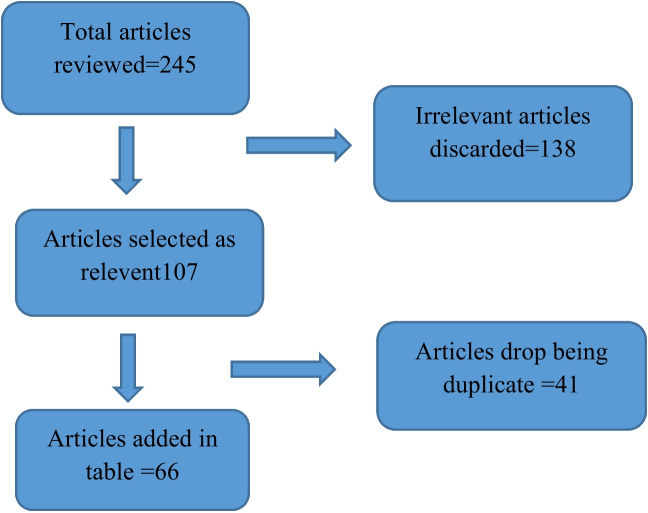
Flow chart showing the main stages of the review process

To find published data on the pharmacology and phytochemistry of anti-diabetic plants, online sources such as Google, Pub Med, Google Scholar, Web of Science, and Flora of Pakistan were utilized. Our Study examined 245 English-language research articles and 138 were then, selected and reviewed based on their pharmacological and phytochemical content. Out of these 138, a total of 66 were included in the review table (Fig. 2). Articles containing irrelevant data not related to our study were dropped. Various keywords, such as the *in-vivo* and *in-vitro* actions of anti-diabetic plants and the activity of secondary metabolites against diabetes were used to search the published literature. Using Tropicos, Plant List, www.flora.org, and the Flora of Pakistan, the accepted family names, recognized plant names, and synonyms were corrected (Table 3).

## Results

### Demographic characteristics of informants

**Fig. 3:**
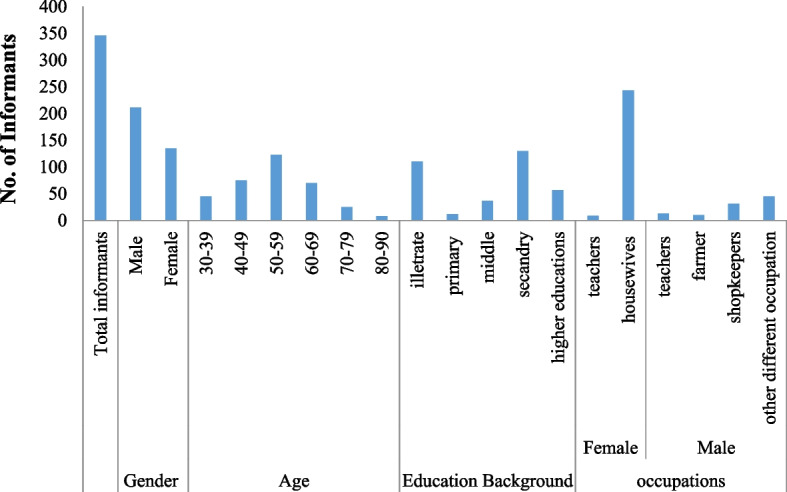
Demographic data of the respondents

A total of 346 respondents (211 male and 135 female) participated in collecting traditional information about the use of monoherbal medicines. They were divided into different modules based on their age, education, and occupation. Most of the primary participants (78%) were aged ≥50 years. Due to having more information about traditional medicinal plants, and recipes used against different diseases as well, males were more involved in commercial activities as compared to females. In males, most of the primary participants were shopkeepers while among females mostly they were housewives. The majority of them completed secondary school education, while the second largest number was illiterate (Fig. 3).

### Quantitative data analysis

#### Use value

The UV of the recorded medicinal plants varied from 1 to 0.03. Species having the highest use value were recorded for *Brassica rapa*, *Melia azedarach* and *Calotropis procera* [1], *Trigonella foenum-graecum* (0.66), *Carica papaya* (0.33), *Abelmoschus esculentus* and *Aloe vera* (0.23). The high used value indicates that these species are highly suggested and well-known by the interviewers, indicating the significance of the species. The plants with less UV are *Apteranthes tuberculata* and *Zygophyllum indicum* (0.03). This least UV specifies that the species have low or few medicinal uses known to the local informants (Table 2).

#### Relative frequency of citation

*Apteranthes tuberculata* was found to have the highest RFC (0.147), followed by *Momordica charantia* (0.11), *Zygophyllum indicum* (0.092), and *Withania coagulans* (0.078) (Table 1). The least RFC was observed for *Brassica rapa*, *Capsicum baccatum*, *Tamarix aphylla*, and *Calotropis procera*, respectively (Table 2).

**Table 1:** ICF of categories of diseases

**S. No**	**Disease Categories**	**Number of species (Nt)**	**Number of use Report (Nur)**	**FIC**
1	Diabetes	38	346	0.89275
2	Sexual	8	40	0.88136
3	Skin	27	195	0.86598
4	Teeth	12	48	0.85714
5	Wounds	15	78	0.81818
6	Respiratory	12	61	0.81667
7	Cardiovascular disorders	15	22	0.33333
Total			790

**Table 2:** Plants of selected areas used for curing diabetes

**Recipes No**	**Botanical Name, voucher number/deposition number, and Family**	**Local Name/Habit**	**Part used**	**Recipe formulation**	**Dosage, treatment duration, and toxicity if any**	**FC**	**RFC**	**UV**	**FL**
1	*Artemisia arborescens* L. BOT-KHS-90/2023-001, Asteraceae	Mashyara/ H	Leaf	2 tablespoons of powder are boiled in 1 cup of water	Take 1 time before breakfast	1	0.003	1	20
				375 grams plant soaked in 4 glasses of water in clay pitcher(recipe will continue for 48 days)	One cup of water twice a day	1	0.003		33.33
2	*Aloe vera* (L.) Burm. f. BOT-KHS-1/2023-001, Asphodelaceae	Zargoy/ H	Leaf	3-4 pieces of gel are eaten	Once a day before breakfast	9	0.026	0.23	43
				A mixture of Aloe Vera gel and honey in crude form	1-2 tablespoons after dinner	2	0.006		17
				The gel of *aloe vera* is used in crude form	Take one before the evening	2	0.006		33.33
3	*Abelmoschus esculentus* Moench.BOT-KHS-20/2023-001, Malvaceae	Bindi/ S	Fruit	Take 4-5 fruits of *Abelmoschus esculentus*, put in 1 glass of water, and leave for the night. Early morning remove the fruit and drink water	Early morning before breakfast	5	0.014	0.2	83.3
4	*Azadirachta indica* A.Juss. BOT-KHS-26/2023-001, Meliaceae	Neem/ T	Leaf	Grind the leaves and make juice from the ground leaves	Half a cup before breakfast. Taking continuously causes increased uric acid	1	0.003	0.3333	25
				Take 10-15 leaves of A. indica ground, put them into 1 glass of water, and leave for the night. After straining through a fine cloth.	Half a cup before breakfast	5	0.014		71.4
5	*Brassica rapa* L. BOT-KHS-91/2023-001, Brassicaceae	Sarsoon/ H	Root	Eaten in salad form	Once a day	1	0.003	1	12.5
6	*Buxus wallichiana* Baill. BOT-KHS-92/2023-001, Buxaceae	Shamshad/ S	Leaf	To reduce water to 1 liter, add 1 Kg of leaves to 1^1/2^ liters of water and put it on fire to dissolve the leaves	One glass in the early morning before breakfast	4	0.012	0.25	50
7	*Capsicum baccatum* L. BOT-KHS-93/2023-001, Solanaceae	Mirch/ S	Fruit	3-4 fruits are eaten in crude form	Once a day	1	0.003	1	50
8	*Cinnamomum sintoc* Blume BOT-KHS-94/2023-001, Lauraceae	Dar cheeni/ T	Bark	Ground into fine powder	One teaspoon is given with tea in the morning and	4	0.012	0.25	57
9	*Calotropis procera* (Aiton) Dryand. BOT-KHS-28/2023-001, Asclepiadaceae	Spalmuy/ S	Flower	4-5 flowers are eaten	Once a day before breakfast	1	0.003	1	12.5
10	*Carica papaya* L. BOT-KHS-95/2023-001, Caricaeae	Papaya/ T	Leaf	Put 2 Kg of Carica papaya leaves in 11 liters of water. After putting the water on a fire, the leaves are dissolved, and the water is reduced to 5 liters.	Half a cup is given 1 time in the morning before breakfast	4	0.012	0.333	30.7
			Fruit	3 pieces of fruit are eaten	Once a day	2	0.006		10.52
11	*Capparis decidua* Edgew. BOT-KHS-30/2023-001, Capparaceae	Karira/ T	Stem	Dry and then grind to make fine powder	One teaspoon before breakfast	2	0.006	0.5	22.22
12	*Citrullus colocynthis* (L.) Schrad. BOT-KHS-5/2023-001, Cucurbitaceae	Kharthuma/ R	Seed	Dried and grinded	Before breakfast in the morning, take half a tablespoon.	8	0.023	0.061	88.88
				4 seeds are eaten in crude form	Before breakfast in the early morning	8	0.023		36.36
			Fruit	Juice of the fruit is used	One teaspoon after each meal, twice a day	2	0.006		33.33
				Fruit is blended in 1 glass of water. Then strained through a fine cloth.	1 glass before breakfast	3	0.009		42.87
13	*Apteranthes tuberculata* (N.E.Br.) KUST.KHS.71/2023-001, Apocynaceae	Pamanai /H	Stem	4-5 pieces of stem are eaten in crude form	Once a day	51	0.147	0.190	94.4
				3-4 pieces with a salt crude form	Before going to bed	1	0.003		33.33
14	*Eriobotrya japonica* (Thunb.) Lindl.BOT-KHS-96/2023-001, Rosaceae	Lokat/ T	Leaf	Dried and then grinded to make powder	1 tablespoon early in the morning before breakfast.1 tablespoon first thing in the morning, just before breakfast. Taking regularly causes stomach problem	1	0.003	1	14.28
15	*Zygophyllum indicum* (Burm.f.) Christenh. & Byng BOT-KHS-50/2023-001, Zygophyllaceae	Spalagza/H	Whole plant	1 kg of the whole plant is added in 1-liter water, leaves for 12 hrs, and after strained through a fine cloth. In 1 liter of water, 1 kg of the entire plant is added. The leaves are then left for 12 hours before being strained through a fine cloth.	Half a cup in the early morning before breakfast	32	0.029	0.0312	94.11
16	*Ficus carica* L. BOT-KHS-52/2023-001, Moraceae	Inzar/ T	Fruit	7-8 dry Fruit are eaten	Once a day	4	0.012	0.25	36.36
17	*Hordeum vulgare* L. BOT-KHS-97/2023-001, Poaceae	Orbasha/ H	Grain	Ground into a fine powder and packed in a capsule	Twice a day before a meal	1	0.003	1	6.66
18	*Launaea nudicaulis* (L.) Hook. f. BOT-KHS-98/2023-001, Asteraceae	Tarisha/ H	Leaf	Dry and then grind to make fine powder	1 teaspoon before breakfast	1	0.003	1	10
19	*Lantana camara* L. BOT-KHS-99/2023-001, Verbanaceae	Camara/ H	Fuit	Fruits are eaten as salad	Once a day	1	0.003	1	16.66
20	*Ligustrum vulgare* L**.** BOT-KHS-100/2023-001, Oleaceae	Amur/ S	Leaf	4-5 leaves are eaten as a salad	Once a day	1	0.003	1	3.70
21	*Moringa oleifera* Lam. BOT-KHS-101/2023-001, Moringaceae	Moringa/ T	Leaf	Dry and then grind to make a fine powder	One tablespoon is given with tea once a day	3	0.009	0.25	9.67
				Leaves shade dried and then ground into fine powder	Half a teaspoon two times before a meal with a little gur	9	0.026		28.12
22	*Momordica charantia* L. BOT-KHS-59 /2023-001, Cucurbitaceae	Karela/ C	Fruit	After blending one fruit with one glass of water, the mixture is filtered using a fine cloth.	1 cup a day before breakfast	8	0.023	0.0983	23.52
				4 cups of water are used to mix the fruits, which are then strained through a fine cloth.	A half cup is given twice a day before the meal.	9	0.026		39.13
				1 fruit is eaten as a salad	Once a day before breakfast	38	0.11		90.43
				Fruits are ground in 1 cup of water	Once a day before breakfast	3	0.009		27.27
				Dry and then grinded	One teaspoon before breakfast	2	0.006		14.28
				Fruits are blended in 3 cups of water after being strained through a fine cloth.	Half a cup is given three times a day, If taken continuously, it causes increased heartbeat	1	0.003		4.76
23	*Melia azedarach* L. BOT-KHS-8/2023-001, Meliaceae	Bakanra/ T	Leaf	An approximate number of leaves are boiled in 1 glass of water	Every morning, before breakfast	1	0.003	1	11.11
24	*Mangifera indica* L. BOT-KHS-102/2023-001, Anacardiaceae	Aam/ T	Leaf	Take some leaves and put them into 2 cups of water. Heat until one cup of water is left	One cup before breakfast	5	0.014	0.2	41.66
25	*Nigella sativa* L. BOT-KHS-62/2023-001, Ranunculaceae	Kalonji/ H	Seed	Take 2 spoon seeds, put them in 1 glass of water, and leave overnight	Take it early in the morning	1	0.003	0.666	5.55
				Grind into fine powder	Half a teaspoon of water 2 times after the meal	2	0.006		16.66
26	*Olea europaea* L.BOT-KHS-65/2023-001, Oleaceae	Zaitoon/ T	Fruit	Soak fruits of Olea in water for a night	4-5 fruit are eaten before breakfast	4	0.012	0.357	21.05
			Fruit oil	Two spoons of olive oil are mixed in one glass of milk	Take early morning	5	0.014		23.80
			Leaf	7-8 leaves are chewed in fresh form	Once a day	3	0.009		13.04
			Leaf	Grind the leaves with water to make juice	Morning 2-3 spoons of juice in one glass of water	1	0.003		5.55
			Bark	5 kg bark is boiled in 15 liters of water. By boiling the water and dissolved bark, reduce the volume to 5 liters	2 spoons before breakfast	1	0.003		9.09
27	*Opuntia anacantha* Speg.BOT-KHS-103/2023-001, Cactaceae	Zakoom/ S	Fruit	4-5 Fruits are eaten in crude form	Once a day	5	0.014	0.2	15.62
28	*Olea capensis* L.BOT-KHS-104/2023-001, Oleaceae	Shown/ T	Fruit	10-15 fruits are eaten in crude form	Once a day	4	0.012	0.428	40
			Bark	1kg bark is boiled in 5 liters of water reducing the water content to 4 liters	2 spoons once a day before breakfast	1	0.003		11.11
			Leaf	4 to 5 leaves are boiled in 1 ^½^ cup and reduced to 1 cup	One time before breakfast	2	0.006		33.33
29	*Psidium guajava* L. BOT-KHS-73/2023-001, Myrtaceae	Amrood/ T	Leaf	250 gm leaves are put in 4-glass water. By boiling reduce it to 2 glasses	1 glass once a day before breakfast	9	0.026	0.1111	34.61
30	*Punica granatum* L. BOT-KHS-105/2023-001, Punicaceae	Anar/ T	Peel	Dry and then grinded into fine powder	1 tablespoon before breakfast	1	0.003	1	12.5
31	*Parthenium integrifolium* L*.*BOT-KHS-106/2023-001, Asteraceae	Lewany bhangy/ H	Flower	5 flowers are eaten in fresh form	Once before breakfast	4	0.012	0.25	30.76
32	*Rhazya stricta* Decne. BOT-KHS-74/2023-001, Apocynaceae	Gandaria/ S	Pod	Dry and then grinded	One tablespoon a day	3	0.009	0.333	27.27
33	*Solanum virginianum* L*.*BOT-KHS-107/2023-001, Solanaceae	Tarha Martha/ H	Seed	Dry and then make fine powder	One tablespoon once a day before breakfast	3	0.009	0.333	15
34	*Tamarix aphylla* (L.) H.Karst. BOT-KHS-83/2023-00, Tamaricaceae	Ghaz/ T	Leaf	Take 3 kg leaves in10 liters of water and leave for 24 hrs. boiled until reduced to one cup of tea	Put three drops of a recipe into one cup and drink before breakfast	1	0.003	1	6.25
35	*Trigonella foenum-graecum* L. BOT-KHS-84/2023-001, Fabaceae	Shamrita/H	Seed	One cup of water and a tablespoon of powder are combined and then left overnight	Take 2 time	2	0.006	0.6666	9.09
				Grinded and to make fine powder	1 teaspoon before breakfast	1	0.003		10
36	*Terminalia chebula* Retz. BOT-KHS-108/2023-001, Combretaceae	Hareer/ T	Fruit	Dry and then grinded into fine powder	One teaspoon before breakfast	1	0.003	1	12.5
37	*Withania coagulans* (Stocks) Dunal. BOT-KHS-87/2023-001*,* Solanaceae	Shapyanga/ H	Fruit	Soaked 5 seeds in 1 glass of water, and left overnight. Remove the water and the seeds are eaten	One early morning	27	0.078	0.0681	87.09
			Leaf	The leaf is grinded to make powder fine powder	One teaspoon before breakfast	2	0.006		13.33
				Extract the juice from the leaves	One cup in the early morning before breakfast	15	0.043		53.57
38	*Ziziphus nummularia* Burm. Wight & Arn BOT-KHS-109/2023-001, Rhamnaceae	Bera/ T	Leaf	Grind some leaves, and put in 1 glass of water. Boiled to reduce half-glass	Once a day before breakfast	4	0.012	0.2	22.22
				7-8 leaves are eaten	once a day	6	0.017		66.66

#### Fidelity level (FL %)

FL indicates the primary participants' choice of a potential plant species to treat a given disease. Fidelity levels ranged from 3.70 % to 94.44 % in the present study. Maximum value of FL (94.44%) was recorded for *Apteranthes tuberculata, Zygophyllum indicum* (94.11), *Citrullus colocynthis* (88.88), and *Abelmoschus esculentus* (83.3) for treating diabetes, skin and wounds. However, *Ligustrum vulgare* (3.70), *Momordica charantia* (4.76), and *Olea europaea* (5.55) had the lowest FL values (Table 2).

#### Informant consensus factor (FIC)

For calculating informant consensus factor (FIC) to various ailment categories 346 use reports were observed for diabetes followed by Skin disorders (195 use reports), wounds (78 use reports), teeth (48 use reports), sexual disorders (40 use reports) and cardiovascular disorders [22] (Table 1). During the present study, it has been noted that diabetes has high Fic value (0.89) followed by dermatological disorders (0.86 each). The lowest value was observed for cardiovascular disorders (0.33).

#### Diversity of medicinal plant species recorded and their taxonomy

**Fig. 4:**
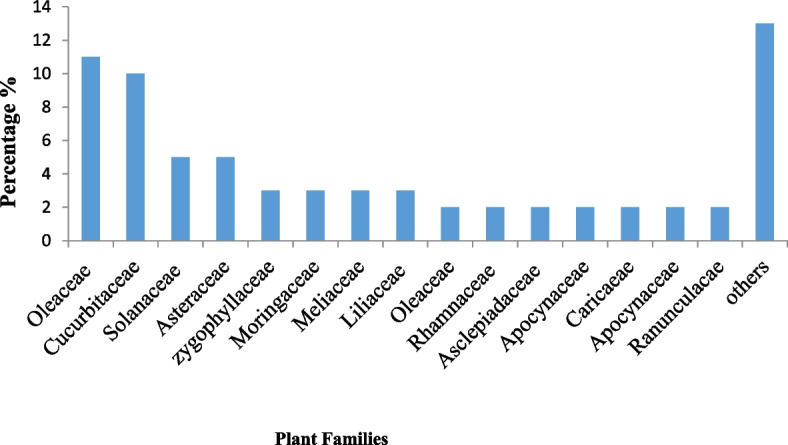
Dominant antidiabetic medicinal plant families of the study area

A total of 38 plant species from 29 families were identified, and local people frequently utilized these to cure diabetes. Most plant species that have been identified are members of the Oleaceae family. The remaining plant species have been divided into the following families: Cucurbitaceae, Solanaceae, and Asteraceae (Fig. 4). Medicinal plant species that have been most frequently cited were *Apteranthes tuberculata*, *Momordica charantia*, *Zygophyllum indicum* and *Withania coagulans* (Table 2).

#### Plant parts used

**Fig. 5:**
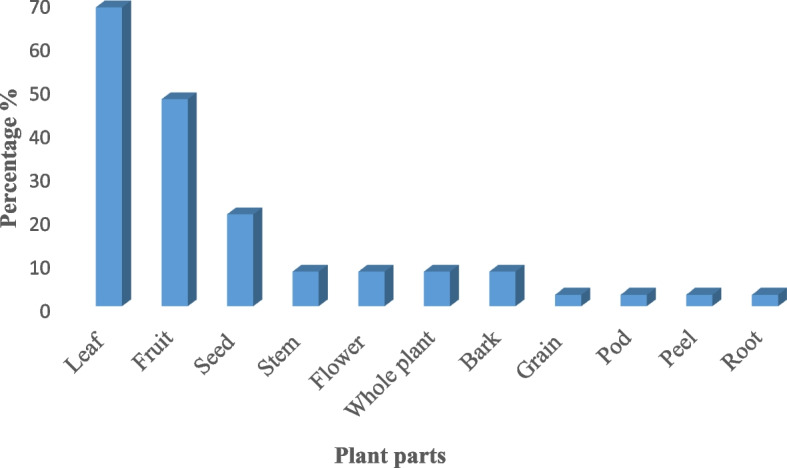
Medicinal plant parts in the herbal formulation against Diabetes

The primary participants in the area mainly utilized leaves, stems, bark, and roots of plants for the manufacture of herbal treatments. The most common medicinal part among the plant parts that have been reported is the leaf, which is followed by the stem, bark, whole plant, seeds, and fruits, in that order (Fig. 5).

#### Habit forms

**Fig. 6:**
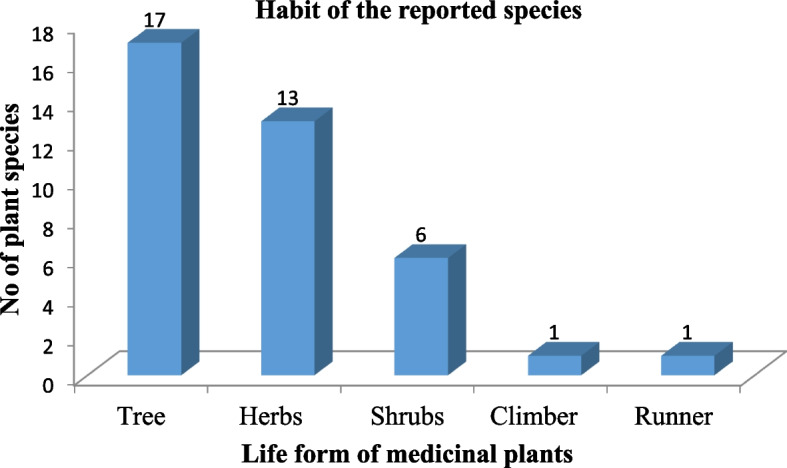
Habits of antidiabetic medicinal plants species

The majority of the medicinal plant species in the study area have been from woody plant species, specifically trees (17 species). They made up as much as 44% of all plant forms. Herbs with (13 species) comprise the second highest followed by shrubs (6 species), climbers, and runners each with 1 species respectively (Fig. 6).

#### Mode of preparation

**Fig. 7:**
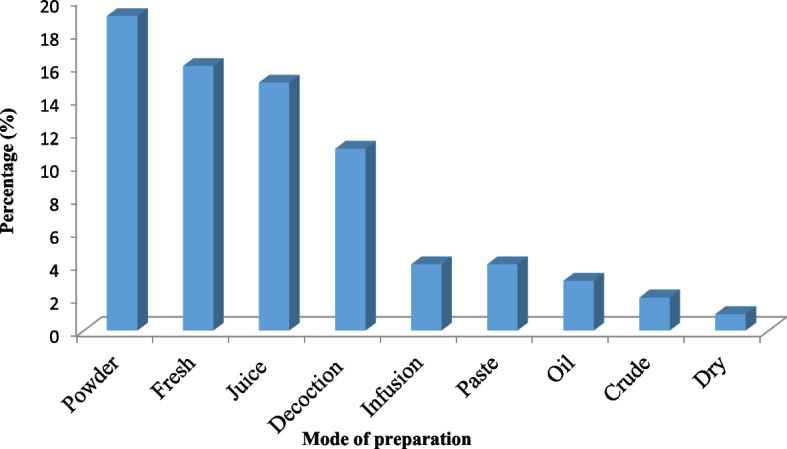
Mode of utilization of medicinal plants

The primary participants most frequently used the following methods to prepare herbal medicines: powder [19], fresh [16], juice [15], and decoction [11] (Fig. 7).

#### Dosage and toxicity

In most cases, the recipes were administered trice and twice a day, at breakfast, lunch, and dinner. Some of these were taken before breakfast, and some after. Most of the primary participants mentioned using traditional remedies after lunch and dinner. In a few instances, toxicity reports have also indicated that long-term usage of *Azadirachta indica* causes a rise in uric acid levels in the bloodstream. *Momordica charantia* and *Eriobotrya japonica* have been linked to difficulties such as elevated heart rate and stomach issues.

#### Review results

Many plant species such as *A*. *vera*, *B. rapa*, *C*. *colocynthis*, *A. tuberculata,* and *C*. *papaya,* etc were analyzed for *in-vivo* antidiabetic activities. For *in-vitro* and *in-vivo* activity, a total of 36 plant species from 28 distinct families were recorded. Thirty-five (35) plant species were evaluated for *in-vivo* animal studies and 28 for *in-vitro* anti-enzymatic activities. Among these families, Oleaceae and Solanaceae were dominant with 4 and 3 plant species followed by Meliaceae and Solanaceae with 2 species each. The rest of the families were reported with one plant species. Different plant parts were found to be utilized for antidiabetic potentials. Leaf as plant part reported mostly followed by fruit and seed. Leaf was used 14 times and fruit 9. Ethanolic, aqueous, and methanolic were among the leading solvents used for the formulation of extracts. The result from the literature also reveals that phenolic, flavonoids, alkaloids, and terpenoids were reported mostly from phytochemical analysis (Table 3).

**Table 3.** Ethnopharmacological evidence of the documented antidiabetic medicinal plants of District Karak, Pakistan

**In vivo**								**In vitro**				
	**Plant name/ Family**	**Part used**	**Extract**	**Dosage (mg/Kg)**	**Route of administration**	**Model**	**Phytocompounds**	**Extract**	**Enzyme**	**Concentration(mg/ml)**	**Inhibition (%)**	**References**
1	*Artemisia arborescens* L./ Asteraceae	Leaf	Ethyl acetate	250	Oral	Wistar rats	Phenolic, flavonoids, tannin	Ethyl acetate	α-amylase	0.9	58.14	[34]
2	*Aloe vera* L./ Asphodelaceae	Leaf	leaf pulp (gel together with latex)	10	Intraperitoneally	Female Albino Rats	Alkaloids, flavonoids, saponins, glycoside, tannins	Methanolic	α-amylase	5	66	[35, 36]
3	*Azadirachta indica* A.Juss./ Meliaceae	Leaf	Aqueous extract	400	Intraperitoneally	Male Wistar rats	Flavonoids, xanthine, terpenoids and glycosides	Ethanol	α-amylase	5	63.5	[37, 38]
									α-glucosidase		66.7	
4	*Abelmoschus esculentus* Moench./ Malvaceae	Fruit	Crude	120	Intraperitoneally	Rats	Tannins, terpenoids, flavonoids	Ethanol	α-amylase	(0.05-2.5	80.2	[39, 40]
									α-glucosidase		93.6	
5	*Brassica rapa* L./ Brassicaceae	Root	Ethanolic	200	Intraperitoneally	Male Wistar rats	Flavonoids, alkaloids, and phytosterols	Ethanolic	α-glucosidase	0.5	44.6	[41–43]
6	*Calotropis procera* (Aiton) Dryand./ Asclepiadaceae	Flower, aerial parts	latex	100 and 400 mg/kg d	Oral	Rats	carboxylic acid, alkenes, and ester	Chloroform	α -amylase	0.2	60	[44, 45]
7	*Citrullus colocynthis* (L.) Schrad./ Cucurbitaceae	Seeds	Aqueous	90	Intraperitoneally	Female Wistar rats	Flavonoids, alkaloids, saponins, tannins, and phenols	Ethanolic	α-glucosidase	0.01	355.5	[46, 47]
		Fruit	Petroleum ether	300&500	Oral	Albino rats	Sugars, fatty acids, flavonoids, alkaloids, glycosides, and essential oils	NA	NA	NA	NA	[48, 19]
8	*Cinnamomum sintoc* Blum / Lauraceae	Bark	NA	NA		NA	NA	Ethanolic	α-glucosidase	1	83.94	[49]
9	*Apteranthes tuberculata* (N.E.Br.)/ Apocynaceae	Whole plant	Ethyl acetate	25& 500	Oral	Rabbits	Steroids, terpenoids, tannins and amino acids;	NA	NA	NA	NA	[29, 50]
10	*Capparis decidua* Edgew./ Capparaceae	Stem	Aqueous and Ethanolic	500	Oral	Albino rats	Alkaloids, carbohydrates, flavonoids, sterols & tannins,	NA	NA	NA	NA	[51]
11	*Capsicum baccatum* L./ Solanaceae	Fruit	Ethanolic	300	Intraperitoneally	Male Sprague-Dawley rats	Oleoresin, phenolic, carotenoids, flavonoids, reducing sugars.	Ethanolic	Ethyl acetate α-amylase	2.5	143.6	[52, 53]
									Hexane α-glucosidase		95.6	
12	*Carica papaya* L./ Caricaeae	Leaf	Ethanolic	100	Intraperitoneally	Wistar mice	Alkaloids, flavonoids, triterpenoids, tannins, and saponins.	Methanolic	α-amylase	0.1	25.2	[54]
13	*Eriobotrya japonica* (Thunb.) Lindl./ Rosaceae	Leaf	Ethanolic	300 mg/kg	Intraperitoneally	Albino rats	Tannins, saponins, alkaloids, flavonoids and carbohydrates	NA	NA	NA	NA	[55]
14	*Zygophyllum indicum* (Burm.f.) Christenh. & Byng/ Zygophyllaceae	Whole plant	Methanolic	150	Intraperitoneally	Rabbits	Saponins, tannins, sterols and triterpenoids, alkaloids	NA	NA	NA	NA	[56, 57]
15	*Ficus carica* L./ Moraceae	Leaves, Fruit	Dichloromethane	500 & 1000	Intraperitoneally	Male mice	NA	Ethanolic	α-amylase	0.1–0.5	104.6	[58, 59]
									α- glucosidase		81.6	
16	*Hordeum vulgare* L./ Poaceae	Grain	Hydro-alcohol	250 & 500	Intraperitoneally	Male Wistar rats	Alkaloids, Flavonoids, Tannins and Saponins	NA	NA	NA	NA	[60, 61]
17	*Lantana camara* L./ Verbanaceae	Fruit	Methanolic	200	Intramuscular injection	Male Albino rats	Carbohydrates, flavonoids, saponins, and fixed oils.	Methanolic	α-amylase	0.1	33.3	[62]
18	*Ligustrum vulgare* L**.** /Oleaceae	Leaf	Aqueous	50-200	Intraperitoneally	Albino rats	NA	NA	NA	NA	NA	[63]
19	*Mangifera indica* L./Anacardiaceae	Leaf	Aqueous	200	Mouth	Albino Rats	Flavonoids, tannins, steroids and terpenoids	Ethanol	α-amylase	0.2	51.4	[64, 65]
20	*Momordica charantia* L./ Cucurbitaceae	Fruit	Ethanol	110	Intraperitoneally	Male albino rats	Alkaloids, flavonoids, tannins, saponins	Ethanolic	α-glucosidase	0.1	0.267	[66, 67]
									α-amylase	0.05	0.298	
21	*Moringa oleifera* Lam./ Moringaceae	Leaf	Ethanolic	250 & 500	Intraperitoneally	Wistar rats	Flavonoids, tannin, cardiac glycosides alkaloids, triterpenoids, saponins,	Ethyl acetate	α-amylase	1	26.3	[68]
22	*Melia azedarach* L./ Meliaceae	Leaf	Ethanol	100	Intraperitoneally	Albino rats,	Polyphenolic, flavonoids, terpenoids, saponins	Ethanolic	α-glucosidase	0.05	72.0	[69, 70]
23	*Nigella sativa* L./ Ranunculaceae	Seed	Ethanol	48	Intraperitoneally	Male albino rats	NA	Hexane	α-glucosidase	0.1	13.1	[71, 72]
									α-amylase	0.2	90.1	
24	*Olea capensis* L./ Oleaceae	Leaf	aqueous	200	Intraperitoneally	NA	Flavonoids, polyphenols, saponins and polysaccharides	Aqueous	α-glucosidase	3.85	81.34	[73, 74]
25	*Opuntia anacantha* Speg./ Cactaceae	Fruit	Aqueous	100	Oral	Male Dawley rats	Flavonoids, alkaloids, steroids and phenols	Hydro-alcoholic	α-amylase	0.5	54.6	[75, 76]
26	*Olea europaea* L./ Oleaceae	Leaf	Aqueous	100, 250 & 500	Oral	Male adult Wistar rats	Glycosides, flavonoids, and poly-unsaturated fatty acids.	Ethanolic	α-amylase	500000	121	[77–79]
									α-glucosidase		165	
		Fruit Oil	Phenolic	20	Intraperitoneally	Male Wistar rats	Phenol, tyrosol, and oxy tyrosol.	Ethanolic	α-amylase	500000	210.5	[80],
									α-glucosidase e		204.3	
27	*Parthenium integrifolium* L*.*/ Asteraceae	Flower	Aqueous	100	Oral	Albino rats	NA	NA	NA	NA	NA	[81]
28	*Psidium guajava* L./ Myrtaceae	Leaf	Methanolic	10	Intraperitoneally	Male Wistar rats	Sugar, tannins, flavonoids, steroids and alkaloids	Methanolic	α-glucosidase	1.0	89.4	[82, 83]
									α-amylase		96.3	
29	*Punica granatum* L./ Punicaceae	Peel	Aqueous	430	Intraperitoneally	Male albino rats	Tannins, steroids flavonoids, quinines, glycosides, cardiac glycosides, carbohydrates	Ethanolic and aqueous extract	α-glucosidase	(0.1–1	53.3,65.48	[84, 85]
30	*Rhazya stricta* Decne./ Apocynaceae	Leaves and fruit	Crude extract	1	Intraperitoneally	Albino rats	Flavonoids, alkaloids, glycosides, saponins, tannins	NA	NA	NA	NA	[86]
31	*Solanum virginianum* L*./* Solanaceae	Whole plant	Aqueous	2000	Intraperitoneally	Albino mice	Alkaloids, glycosides, proteins and amino acids, flavonoids, and saponins	NA	NA	NA	NA	[87]
32	*Tamarix aphylla* (L.) H.Karst./ Tamaricaceae	Leaf	Methanolic	400	Intraperitoneally	Male Wistar rats	Flavonoids, cardiac glycosides, steroids and terpenoids	Methanolic	α-glucosidase	0.024	67	[88, 89]
33	*Trigonella foenum-graecum* L./ Fabaceae	Fruit	Ethanolic	200	Oral	Male albino Wister rats	Tannins, phenols, terpenoids	Petroleum ether	α-amylase	1	65.9	[90, 91]
34	*Terminalia chebula* Retz./ Combretaceae	Seed	Ethanolic	100, 250, & 500	Oral	Male Wister rats	Polyphenols and flavonoids	Ethyl acetate	α-amylase	0.25	64.5	[92]
									α-glucosidase		52.5	
35	*Withania coagulans* (Stocks) Dunal./ Solanaceae	Fruit	Aqueous	250	Intraperitoneal Injection	Male albino Wistar rats	Carbohydrates, glycosides, saponins, phenols, tannins, alkaloids and flavonoids	Ethanolic	α- glucosidase	0.05	45.4	[78, 93]
36	*Ziziphus nummularia* Burm.Wight & Arn/ Rhamnaceae	Leaf	Ethanol Aqueous	250 & 500	Oral	Swiss albino Male Wister rats	Alkaloids, flavonoids, saponins, mucilage, tannins, steroids and phenolic compound	Saponin Extract	α-amylase	0.16	88.3	[94, 95]

*In-vitro* antidiabetic activity reveals that *C. colocynthis* and *O. europaea* showed the highest zone of inhibition against a-amylase (210%) and a-glucosidase (355% and 204%) respectively. *C. baccatum* against a-amylase showed a 143% zone of inhibition while *F. carica* with 104% (Table 3). However, two plant species reported in the survey that is *Buxus wallichiana* Baill and *Launaea nudicaulis* (L.) found to have rare data regarding *in-vivo* and *in-vitro* anti-diabetic activities.

## Discussion

The present study revealed that people who are local inhabitants have great traditional knowledge regarding plants having medicinal potential. The study was based on traditional uses of plants used against diabetes by local people. Data revealed that people preferred plants over modern drugs to treat diabetes. The plants are safe and productive.

The study was conducted randomly from different areas of District Karak to explore that not only local healers used these plants but also common people who did not practice medicine have great knowledge of plants having anti-diabetic properties. In the present study total of 346 respondents (211 male and 135 female) participated in collecting usual information about the use of herbal medicines. The data was divided into different modules based on their age, education, and occupation. According to [96, 97], a total of 67 informants out of 103 individuals (response rate: 69%) from nine ethnic groups were interviewed; 70% of the informants were men and 30% were women. A study was conducted by [98] and reported a total of 150 respondents (100 females and 50 males). They participated while collecting information regarding multiple diseases including diabetes, gastrointestinal, and urinary tract infections. Our results agreed with [99] where males were the major respondents.

The results of the present study show that the maximum use values (UV) were noted for *Brassica rapa*, *Melia azedarach,* and *Calotropis procera* [1] while *Apteranthes tuberculata* (0.147) followed by *Momordica charantia* (0.11), *Zygophyllum indicum* (0.092) and *Withania coagulans* (0.078). In 2019 [100] applied UV and RFC on the collected data and reported the highest RFC for *Adiantum venustum* (0.27) used in the form of paste for wound healing properties, *Artemisia fragrans* (0.25) used in the treatment of boils, similarly *Aconitum chasmanthum* (0.24) used as a decoction for treatment of mumps and measles. The UV recorded highest for *Pisum sativum* (0.143), *Cynodon dactylon* (0.125) and *Adiantum venustum* had a very low use value (0.021).

Among all modes of preparation, powder was most preferable. The results of the study conducted in Northern Pakistan by [100] reported that in various preparation methods, the powder was most frequently used.

According to the current study, the percentages of plant parts that are used are as follows: 68% for leaves, 47% for fruits, 21% for seeds, 7% for flowers and whole plants, and 4% for rhizomes and areal stems with branches. According to a study by [101] leaves (90%) fruits and roots (16%), seeds, and entire plants (8%), each. This is beneficial in terms that plants may not be damaged as leaves were removed from plants rather than stem and root. In another study, the most common ingredient utilized to prepare traditional medicines was leaves. Rich in several phytochemicals, including tannins, glycosides, alkaloids, and saponins, leaves are readily available [102].

Trees and Herbs were major life forms of plant species used as antidiabetic medicine. These findings were in accordance with many other surveys documented for monoherbal recipes where trees and herbs were the dominant plant status [103, 104]. It may be because traditional healers have access to a large number of trees or herbs that are natural.

In the current survey, 38 plant species belonging to 29 families were recognized which were frequently used by inhabitants to treat diabetes. Among these 29 families, four families Oleaceae, Cucurbitaceae, Solanaceae, and Asteraceae were dominant. According to [105] families that were frequently cited are Solanaceae and Moraceae followed by Apiaceae, Cucurbitaceae, Euphorbiaceae, and Fabaceae. The remaining families contributed a minor role in the ailment of diseases traditionally.

The species that the inhabitants preferred to treat specific diseases based on their level of fidelity. There were significant variations in the documented species' fidelity levels for a specific disease. Among the species that have been reported, a maximum value of FL (94.44%) was recorded for *A. tuberculata, Z. indicum* (94.11), *C. colocynthis* (88.88), and *A. esculentus* (83.3) for treating diabetes, skin and wounds. On the other hand, the least FL was recorded for *L. vulgare* (3.70), *M. charantia* (4.76) and *O. europaea* 5.55 respectively.

According to a study conducted in 2023, *Berberis lycium* had the highest FL value (89.9%) when used to treat ulcers and stomach pain*. Decaspermum blancoi* came in second with an FL value of 62.2 percent when treating diarrhea and abdominal pain, and *Sageretia thea* and *Solanum nigrum* each had an FL value of 53.3% when treating hepatitis and blood purification, respectively. However, the *Xanthium strumarium*, *Cephalanthera longifolia,* and *Astragalus grahamianus* showed the lowest fidelity level [106].

FL ranged from 28 to 100%. The plant species mostly used in the study area with 100% fidelity level were *Aesculus indica, Amaranthus viridis, Artemisia scoparia, Cedrus deodara, Chenopodium botrys, Jasminum humile, Malva sylvestris, Layia chrysanthemoides*, *Thalictrum foliolosum*, and *Urtica dioica* [107].

In the present study, the ICF values for different ailment categories treated by the local informants in this survey ranged from 0.33 to 0.89. Diabetes, skin, wounds, and respiratory 0.89, 0.86, and 0.81 ICF, respectively, were ranked as the most popular ailment categories for medicinal plants in this region. Diabetes disorder scored the highest ICF (0.89). This unexpected result is due to the high use report of few medicinal plants for treating diabetes mellitus such as *Apteranthes tuberculata, Zygophyllum indicum, Citrullus colocynthis*, and *Abelmoschus esculentus* with 346, 195, 78, and 61 use reports, respectively.

The condition with the greatest ICF was diabetes (0.92). This surprising outcome can be attributed to the high number of reported uses of a select few medicinal herbs, such as *Tecomella undulata, Berberis integerrima*, and *Citrullus colocynthis*, which had 91, 61, and 41 usage reports, respectively, for treating diabetes *mellitus*. In this instance, the antidiabetic qualities of C. colocynthis, a well-known medicinal plant in the province of Kerman, are well documented [108].

These findings are due to high-use reports for plant species such as *Calotropis procera, Pergularia tomentosa, Rhazya srticta,* and *Tecomella undulata* in the treatment of eczema, wound healing, and other skin disorders]. A different study found that endocrine problems had a high FIC value (0.90), followed by fever (0.88), gastrointestinal disorders, and dangerous bites. The dental care category has the lowest value (0.60) [109].

Compounds reported in *A. tuberculata* such as pregnane glycosides, and flavone glycosides have anti-diabetic properties [110]. Bioactive polysaccharides such as rhamnose and galacturonic acid belong to the class carbohydrates found in *A. esculantus* (okra) and reportedly have biological functions in the body [111]. Another study [112] reported compounds like pectin, guar gum, and carboxymethylcellulose (CMC) add good results in various conditions like hyperlipidemia and diabetes. Saponin a natural product found in *C. colocynthis* has the anti-hyperglycemic effect and may interact with many metabolic pathways or insulin metabolism. It also affects glucose homeostasis directly or indirectly [113]. A compound isolated from *M. charantia* L. named charantin is documented to have insulin-like activity by promoting the release of insulin and decelerating glucogenesis [114].

Numerous plant species, including *A. vera, B. rapa*, *C. colocynthis*, *A. tuberculata*, and *C. papaya,* among others, were tested for their in-vivo antidiabetic properties, according to the review results. For *in-vivo* and *in-vitro* activity, a total of 36 plant species from 29 distinct families were described. For *in-vivo* animal research, 35 plant species were assessed, and 28 were investigated for their in vitro anti-enzymatic properties. With four and three plant species, respectively, Oleaceae and Solanaceae were the most numerous of these families, followed by Meliaceae and Solanaceae with two species apiece. A study by [115] found that 255 plant species from 70 families were recorded. It was claimed that the two most represented families are Compositae and Lamiaceae. Most Moroccans use *Artemisia herba-alba*, *Nigella sativa*, *Olea europaea*, *Marrubium vulgare*, *Trigonella foenum-graecum*, and *Allium cepa* as common plant species.

## Conclusions and recommendations

Having assessed, the ethnopharmacological properties of antidiabetic medicinal plants has provided valuable insights into the traditional diabetes management practices in the study area. The present study highlights the importance of indigenous wisdom in preventing diabetes and identifies several plants that may have anti-diabetic properties. The findings of our study bridge the gap between traditional and modern medicine, encouraging further investigation and validation of these plants' therapeutic effects through in-vivo, in-vitro, and phytochemical activities.

Validating the anti-diabetic properties of identified medicinal plants requires rigorous scientific research. For instance, phytochemical analysis and bioassays may be conducted to understand the active compounds causing the observed effects. Furthermore, the findings of this study support the initiation of clinical trials to examine the safety, efficacy, and optimal dosage of the most promising antidiabetic herbs. The study proposes standard protocols for preparing and administering herbal remedies to ensure consistency and quality control. Moreover, it is also important to promote the cultivation of medicinal plants to reduce pressure on wild populations and ensure a sustainable supply.

To summarize, ethnopharmacological assessment of antidiabetic medicinal plants offers significant potential for diabetes management. By respecting traditional knowledge, conducting rigorous research, and implementing appropriate regulatory measures, we can harness the potential of investigated medicinal plants and improve diabetes care.

## Data Availability

The article contains all the data generated or analyzed during this study. Upon request, we will make the materials and data of our study available to other researchers from the principal author (Ms. Amina Nazar amnanazarktk@gmail.com).
